# Defense Pathways of Wheat Plants Inoculated with *Zymoseptoria tritici* under NaCl Stress Conditions: An Overview

**DOI:** 10.3390/life14050648

**Published:** 2024-05-20

**Authors:** Behzat Baran, Fatih Ölmez, Beritan Çapa, Murat Dikilitas

**Affiliations:** 1 Plant Protection Research Institute, Sur, Diyarbakır 21110, Türkiye; behzatb@hotmail.com; 2 Department of Plant Protection, Faculty of Agriculture, Sivas University of Science and Technology, Sivas 58010, Türkiye; fatiholmez@hotmail.com; 3 Department of Plant Protection Şanliurfa, Faculty of Agriculture, Harran University, Sanliurfa 63000, Türkiye; brtncapa@gmail.com

**Keywords:** cereals, wheat, salinity, pathogen, combined stress, breeding

## Abstract

Due to being sessile, plants develop a broad range of defense pathways when they face abiotic or biotic stress factors. Although plants are subjected to more than one type of stress at a time in nature, the combined effects of either multiple stresses of one kind (abiotic or biotic) or more kinds (abiotic and biotic) have now been realized in agricultural lands due to increases in global warming and environmental pollution, along with population increases. Soil-borne pathogens, or pathogens infecting aerial parts, can have devastating effects on plants when combined with other stressors. Obtaining yields or crops from sensitive or moderately resistant plants could be impossible, and it could be very difficult from resistant plants. The mechanisms of combined stress in many plants have previously been studied and elucidated. Recent studies proposed new defense pathways and mechanisms through signaling cascades. In light of these mechanisms, it is now time to develop appropriate strategies for crop protection under multiple stress conditions. This may involve using disease-resistant or stress-tolerant plant varieties, implementing proper irrigation and drainage practices, and improving soil quality. However, generation of both stress-tolerant and disease-resistant crop plants is of crucial importance. The establishment of a database and understanding of the defense mechanisms under combined stress conditions would be meaningful for the development of resistant and tolerant plants. It is clear that leaf pathogens show great tolerance to salinity stress and result in pathogenicity in crop plants. We noticed that regulation of the stomata through biochemical applications and some effort with the upregulation of the minor gene expressions indirectly involved with the defense mechanisms could be a great way to increase the defense metabolites without interfering with quality parameters. In this review, we selected wheat as a model plant and *Zymoseptoria tritici* as a model leaf pathogen to evaluate the defense mechanisms under saline conditions through physiological, biochemical, and molecular pathways and suggested various ways to generate tolerant and resistant cereal plants.

## 1. Introduction

Under both abiotic and biotic stress conditions, crop plants exhibit various defense responses. Pathogen attack activates the immune system of plants, depending on the capacity of the plants and the virulence of the pathogens. The production of antimicrobial compounds, reinforcement of cell walls, and activation of defense-related genes, enzymes, and other biochemical compounds could occur upon the attack of pathogens. On the other hand, nutrient imbalance, osmotic pressure, reduction in water potential, and ion toxicity could be observed in plants because of abiotic stressors. Water uptake and maintenance could be interrupted. In general, plants activate antioxidant defense mechanisms in abiotic or biotic stress conditions; however, coping with two types of different stresses under combined stress conditions could be very difficult, even if a plant becomes highly resistant to one of those stress factors. Plants should show specific responses to tolerate both stress factors; however, the duration and strength of the stress are quite crucial under these circumstances. When plants are exposed to multiple stresses simultaneously or sequentially, such as a combination of pathogen infection and salinity stress, the responses of plants become very complex and interconnected. In such cases, the interaction between the stressors may be additive, synergistic, or antagonistic. These interactions could be reflected as more severe symptoms of pathogens or salinity in plants in additive or synergistic cases [1]. We even observed unique stresses that are not characteristic of individual stress factors. In some cases, symptoms that are characteristic of a particular stress factor may be less expressed or not visible if the interactions are antagonistic. In such cases, one of the stress agents might exert priming effects on plants. For example, we observed that different stresses could lead to better defense responses if negative interactions could occur between stressors. Stresses of different characters (abiotic or biotic) on plants might “crosstalk” using the same metabolic or defense pathways as well as sharing the same signaling networks. For example, Satapathy et al. reported aluminum (Al) toxicity at various concentrations (0, 50, 100 µmol L^−1^) and *Fusarium incarnatum*-*equiseti* infections in *Cajanus cajan* (L.) Millsp [2]. The plants experienced a significant increase in reactive oxygen species (ROS) generation, cell death, and other antioxidant enzymes, such as superoxide dismutase (SOD), catalase (CAT), and ascorbate peroxidase (APX). However, at 50 µmol L^−1^ Al conditioning, a significant counteraction against *Fusarium* infection was evident, which was marked by a decrease in ROS and cell death in the *C. cajan* plants. However, in most cases, pathogen infection or abiotic stress can weaken the defense mechanisms of plants, making them more vulnerable to secondary stress. Very few studies have mentioned that abiotic stresses, such as drought, heat, and salinity, could enhance the defense mechanisms of plants against pathogen attacks by activating biochemical metabolites through gene expression [3].

In general, pathogens trigger the production of ROS and reactive nitrogen species (RNS), causing oxidative stress and leading to physiological, biochemical, and molecular modifications in cells. Abiotic stress factors such as salinity, drought, heat, cold, and environmental pollution also result in the production of ROS and RNS in cells. If a pathogen and an abiotic stress factor occur simultaneously or sequentially in a plant ecosystem, severe stress occurrence is inevitable, even if the crop plant is generated to be resistant to one stress group. Under these circumstances, plants tend to increase their antioxidant molecules to cope with the negative effects of ROS and RNS to the best of their capacity. Crop plants, in general, tend to follow a different pattern under stress conditions, e.g., they could produce fewer seeds or fruits to survive; however, if they are exposed to multiple stressors, the production of seeds or fruits is significantly reduced and the quality deteriorates [4]. If a crop plant is moderately resistant or tolerant to either biotic or abiotic stress factors, it may survive and produce seeds or fruits, depending on the severity or duration of the combined stress factors.

Abiotic and biotic stresses do not always come with the increase in global warming, environmental pollution, and population growth; sometimes, inappropriate agricultural practices such as overirrigation or overfertilization and the selection of inappropriate cultivars for crop production could create additional stress when combined with biotic stress agents. For example, the application of overfertilization could trigger sporulation or mycelial growth of fungi. We noticed that fertigation containing Cl^−^, Na^+^, Ca^2+^, Mg^2+^, and K^+^ ions on the surface of wheat leaves led to the synthesis of microproteins and carbohydrates in *Zymoseptoria tritici*, the leaf-infecting fungal agent, which led to the breakdown of thin water films on the surface of the leaves. When the water surface tension broke up due to the involvement of proteins and carbohydrates, *Z. tritici* was carried away from the point of establishment to the vicinity of the stomata. This hastened penetration through the stomata (unpublished data). We noticed that the pathogenicity and spread of the fungus increased upon fertigation in the presence of a water film on the leaf surface. In general, pathogens are not able to quickly develop mycelia or conidia under high NaCl conditions (e.g., 150 mmol L^−1^ NaCl); however, sporulation becomes abundant for soil-borne and leaf-infecting pathogens at low NaCl concentrations (e.g., 50 mmol L^−1^ NaCl) [5]. High salinity, such as 100 mmol L^−1^ NaCl and above, creates an unfriendly environment for leaf-infecting or soil-borne pathogens in terms of development and sporulation; however, tolerance to salinity is generally high in microorganisms compared with crop plants in the same environment [6,7]. It is clear that an increase in water pressure due to increases in the NaCl concentration decreases the water potential and puts the pathogen under stress. Under these circumstances, pathogens should not easily infect the host through the soil or leaf stomata, depending on the position of the fungi and the concentration of NaCl, which restricts the movement of the fungi. However, recent findings have shown that proteins and carbohydrates of pathogens break up the water film layer when triggered via ions. For example, the osmotic stress conditions created by 0.4 mol L^−1^ NaCl or KCl or 0.6 mol L^−1^ sorbitol on *Setosphaeria turcica*, a causal agent of northern corn leaf blight disease in maize, sorghum, and related grasses, decreased the mycelium growth rate of the fungus and resulted in the production of vesicular structures and flocculent outside the hyphal cell wall [8]. The infecting fungus quickly grew in the vicinity of the stomata and penetrated it directly without facing any defense barriers. The authors reported that osmotic stress quickly activated the HOG-MAPK pathway and upregulated the stress-related genes within 30 min of exposure to osmotic stress. They found that the germination rate and yield of conidia were encouraged under osmotic stress. The stress-related gene *StFPS1*, which is involved in the formation of appressorium and penetration peg, facilitated the penetration ability of *S. turcica*, thus increasing the pathogenicity of the conidia. The authors also observed that the germination time of the conidia was shortened. They established a mechanism by which a quicker osmotic response of the fungus was closely related to its more aggressive pathogenicity.

If we understand the mechanisms and consequences of combined stress, we could fertilize the leaves when the fungus is absent or in its inactive stages. Because pathogens of any kind (saprophyte, facultative, obligate, necrotroph, or semi-biotroph) on leaves could be mobilized and motivated with thin water film containing ions and can secrete extracellular proteins and metabolites that would break the tension of the water film, we could prevent the spread of pathogens by modifying the application time of fertilizers. Otherwise, semi-biotroph pathogens such as *Z. tritici* could propagate and easily reach the stomata [9]. Although obligate pathogens cannot extend their mycelia to reach the stomata, mobilization of these pathogens should not be ignored, and more studies should be performed to evaluate the pathogens under low and high NaCl conditions. Via small modifications to the leaf surface, we could control the pathogens and save our budget and energy with little effort, which would otherwise be spent on chemicals for the control of these pathogens.

From our perspective, we evaluated cereal plants, particularly wheat, under pathogen and salinity conditions. Salinity and disease interactions had previously been evaluated in many crop plants in which salinity and root pathogens were considered at first. Defense mechanisms have been investigated from this perspective. However, leaf pathogens and salinity interactions are as important as other interactions. Here, two different routes, one “salinity route” through the xylem and the other “pathogen route” through the phloem, in the leaves severely threaten the defense mechanisms via different pathways. Vascular bundles (sieve tube elements) in wheat (monocotyledons) also create complexity in the defense mechanisms.

In this review, we evaluated pathogen, abiotic stress (mainly salt), and plant (wheat) interactions from various perspectives.

## 2. Abiotic Stresses in the Cereal Growth Environment

The significance of wheat, a vital component of global nutrition and human health, continues to grow. However, the areas of cultivation are diminishing. Environmental pollution, soil salinity, global warming, floods, excessive rainfall, etc., have a substantial impact on wheat production. Despite efforts to increase yields per unit area to close the gap between the consumption of wheat products and population growth, the desired success has not been achieved. Success in increasing crop production remains elusive because of the involvement of disease factors under abiotic stress conditions. The combination of stress factors not only reduces crop yields and results in economic losses but also diminishes crop quality and poses a threat to human health due to the consumption of low-quality products. Therefore, it is imperative to implement measures to mitigate the impact of these stress factors on crop yields. This is corroborated by the latest report published by the International Agricultural Council (IAC), which highlights the decline in global wheat production [10].

On a global scale, 728.9 million tons of wheat (*Triticum aestivum* L. and *T. durum* D.) were produced on 221 million hectares [11]. Globally, most cultivation is performed on semi-arid lands. In general, more than half of the daily calorie intake is derived from wheat and its derivatives. High adaptability, simplicity of production, and ease of transport, storage, and processing may be the primary reasons for this proportion.

The world population is projected to reach 9.7 billion in 2050 and 10.9 billion in 2100 [12]. Annual cereal production should increase by at least 30% to meet the food needs of the growing population. Given that agricultural land has reached its limits, increasing wheat yields will require either bringing marginal lands into production or increasing crop production per unit area. In terms of wheat production, the European Union countries rank first with a share of 19%, while China and India rank second and third with 17% and 11%, respectively [13].

Abiotic stresses, such as salinity, drought, cold, and high temperatures, are significant factors that can reduce both the quality and quantity of crop yields by limiting water uptake, disrupting ionic balance, and causing toxicity at high concentrations. This results in various physiological, morphological, biochemical, and molecular changes in the plant. Current climate projections suggest that over the next 50–100 years, temperature increases will be prevalent in the interior regions of Central Africa and Europe. This would lead to shorter production seasons, increased threats of salinization and desertification due to rising sea levels, and a decrease in land suitable for agriculture [14,15]. Climate change can not only reduce crop yields and alter their nutritional value but can also impact the habitats of plant pests and pathogens, altering their characteristics [16,17].

## 3. Responses of Cereal Plants to Saline Stress

Soil salinity is the most commonly observed stress in arid and semi-arid climates [18]. In these regions, salinization occurs more rapidly with irrigation. During irrigation, salt in the lower layers of the soil is drawn upward through capillary action via evaporation and accumulates in the root zone. Other causes of salinization include improper irrigation, inadequate drainage, and high levels of soluble salts in the irrigation water. It is estimated that approximately 30% of sustainable agricultural land will face salt stress in the next 25 years, and this figure is expected to rise to 50% by the end of the 21st century due to the possible expansion of salt-affected areas [19,20].

Every year, 10 million hectares of land around the world become unusable due to salinity problems [21]. According to the latest records, 20% of the 230 million ha of agricultural land is affected by salinity to varying degrees [22]. For the above reasons, more than 800 million hectares of arable land worldwide are affected by salinity [23]. Salt stress causes many symptoms, such as stunting, inhibition of plant growth, delayed opening of buds and fruits, shortening of shoots, shrinking of leaves, necrosis, wilting, and cell death. It also disrupts the hormonal balance, reduces quality, and predisposes plants to other stresses. When plants are exposed to salinity, they respond to stress stepwise. This consists of different stages, such as the recognition of stress, the adaptation phase, the repair phase, and the defeat phase. First, the roots are triggered by the presence of excess salt ions, followed by a series of molecular, biochemical, and physiological reactions. The plant prepares itself for defense against salt stress via modulation of gene expression at the transcriptional level. Physiologically, closure of the stomata and accumulation of osmoprotectants are the primary responses to maintain water homeostasis [24]. Ion transport should be regulated via the exclusion or storage of ions in vacuoles to maintain cellular homeostasis in the crucial parts of plant cells to minimize ionic toxicity [25]. In addition to these responses, the production of ROS and RNS is triggered [26]. The production of free radicals such as superoxide ions (O_2_ˉ), hydroxyl radicals (OH), singlet oxygen (ˡO_2_), and hydrogen peroxide (H_2_O_2_) destroys metabolic activities during growth and development [27]. At low concentrations, these molecules act as signaling molecules involved in hormonal maintenance and plant defense mechanisms [19,25]. However, high concentrations of these molecules cause cell membrane damage, protein oxidation, DNA lesions, irreparable metabolic dysfunction, cell death, and the activation of programmed cell death [28]. However, plants have evolved antioxidant defense systems to counteract the harmful effects of ROS and RNS. Enzymes such as SOD, CAT, peroxidase (POX), and APX play crucial roles by regulating and scavenging free radicals and converting them into non-harmful forms to maintain the oxidative balance under salt stress [29]. For example, SODs in plants are classified into three different groups: the first group is copper/zinc (Cu/Zn)-SOD, the second group is manganese (Mn)-SOD, and the last group is iron (Fe)-SOD. They are all distributed in different cellular compartments. Cu/Zn-SODs are mainly found in chloroplasts, cytosol, and mitochondria, Mn-SODs in mitochondria, and Fe-SODs in chloroplasts, peroxisomes, and mitochondria [30].

Additionally, non-enzymatic antioxidant substances, including proline, glycine betaine, arginine, glucose, vitamins, and various metabolites, act as osmoprotectants, stabilize macromolecules, and are involved in maintaining proper cellular function under saline conditions [31]. Research has shown that the synthesis of these metabolites or external application to plants can reduce the stress level [32]. At the molecular level, upon perceiving salt stress, specific genes are activated or repressed, leading to the synthesis of stress-responsive proteins, which play a vital role in protecting plants against the harmful effects of salt stress [33]. One of the key transcription factors involved in the response to salt stress is *DREB1A*. This transcription factor binds to specific DNA sequences and activates stress-related gene expression. Its overexpression has been shown to enhance salt tolerance in various plant species, including wheat [34]. Öztürk and Demir reported that NaCl molecules disrupt the hydrogen bonds of proteins in the cell membrane and result in the release of ions such as K^+^ and Ca^2+^ in and out of the cell environment [35].

There is a clear difference between salt-sensitive and salt-tolerant varieties of crop plants in terms of the salt response. Mandhania et al. exposed 4-day-old seedlings of salt-tolerant KRL-19 and -sensitive WH-542 wheat varieties to 50 and 100 mmol L^−1^ NaCl stress and reported that the sensitive variety had lower water content and a lower K^+^/Na^+^ ratio. The level of lipid peroxidation and H_2_O_2_ accumulation was higher in the sensitive variety because of membrane damage [36]. Similar issues were reported by Hu et al., who compared the salt-tolerant Quickstart II genotype with two salt-sensitive *DP1* genotypes of perennial ryegrass (*Lolium perenne*) [37]. Hasanuzzaman stated that proline is a dominant metabolite for osmotic regulation and does not alter the pH level in the plant because of its high solubility in water and low molecular weight, unlike previous reports [22]. He stated that the *OsSALP1* gene, which regulates salt stress in wheat plants, encoded a specific protein in membranes involved in the formation of free proline by upregulating the level of the *OsP5CS* gene.

Proteins are of great importance for the stress response because they are directly involved in stress tolerance. It is evident that salinity increases many stress-related proteins (ion transporters, ROS-scavenging enzymes, and dehydration-induced proteins) and leads to complex adaptations in gene expression, cell signaling, and cellular metabolism. Maršálová et al. and Dissanayake et al. stated that proteomic analysis of root tips of the wheat cultivar Scepter expressed many proteins related to carbon and energy metabolism after exposure to 150 mmol L^−1^ NaCl stress after 6 days [38,39]. The upregulation of proteins involved in sugar and amino acid metabolism was found to be directly related to the energy metabolism of plants. An upregulated protein system also reorganizes cellular metabolism under stress. However, when salt stress exceeds the tolerance limit of the plant, it can permanently disrupt protein metabolism, as evidenced by the reduction of the soluble protein content of the grain in barley [40].

Transcription levels have been assessed in plants exposed to salt stress in recent studies. Amirbakhtiar et al. identified 4290 differentially expressed genes (2346 upregulated genes and 1944 downregulated genes) out of 98,819 genes with 26,700 copies in a salt-tolerant wheat cultivar (Arg) following salt stress. The copies were found to be related to phenylpropanoid synthesis, hormone signaling, and MAPK signaling mechanisms [41]. They concluded that the copying and gene regulation process was higher in the resistant variety than in the susceptible one. He et al. compared miRNA and mRNA sequences of a salt-tolerant wheat variety (Qing Mai 6) under salt stress and normal conditions and identified 8 miRNAs and 11 mRNAs. The miRNAs were involved in regulating stress-related genes, antioxidant mechanisms, nutrient uptake, and lipid metabolism [42].

Not only are physiological, biochemical, and molecular pathways modified but also anatomical and morphological structures are affected when plants are exposed to salt stress. This increases the predisposition of plants to other abiotic or biotic stresses. For example, Nassar et al. measured the carbon fixation of wheat plants grown under salt stress in the range of 0–12.0 dS/m and determined the anatomical differences in the leaf [43]. Decreases in the leaf diameter, leaf thickness, number of vascular bundles, and phloem and xylem diameters were evident. It is evident that the roots are the most affected parts under saline conditions. They may, however, exhibit a shallower and denser root system for efficient water and nutrient uptake from the top parts of the soil [44].

**Figure 1:**
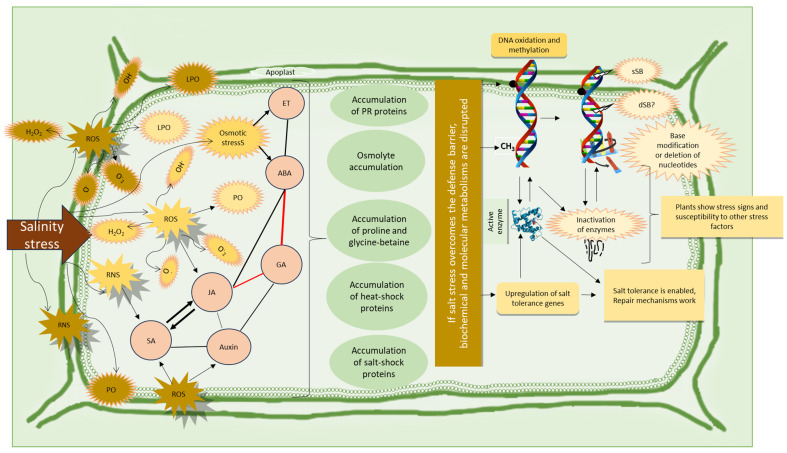
Overview of the effects of salinity stress on cell metabolism.

The salt tolerance of wheat over the years has increased from 60 to 80 mmol L^−1^ NaCl to much higher levels in many commercially available cultivars (ca. 150 mmol L^−1^ NaCl) [45]. However, in recent years, the duration and severity of stress and its interactions with other abiotic and biotic stresses have threatened newly developed plant varieties [46]. We will evaluate this in the following sections. Here, we briefly illustrate the effect of salinity, e.g., NaCl, on the root and shoot cells of wheat in Figure 1. The effects of the combined stress of salinity and disease interactions are evaluated in the next section (Section 6).

When plant cells are exposed to low levels of salinity stress, signaling mechanisms, through specific proteins, the Ca^2+^ transduction pathway, and ROS (at very low concentrations), induce gene expression [24,47]. Under salinity stress, mitochondria and chloroplasts work hard to improve the conditions of plants; however, as a side effect, the ROS levels increase rapidly because these sites are characterized by ROS production [3,26,38]. Osmotic stress and ion imbalance also lead to ROS production. ROS have two mechanisms, one at low concentrations that could act as signaling molecules and the other at high concentrations that could act as stress molecules [22,28]. However, a high level of ROS results in the degradation of proteins (especially chlorophylls) and modifies pigment composition, enzymes, and DNA molecules [27]. As a result of this, membranes are highly damaged, solutes in the cell are lost, viscosity decreases, water moves slowly in the cell, and communications are impaired and lost in severe cases. Metabolic activities are significantly impaired. Although antioxidant enzymes reduce the toxic effects of ROS, prolonged stress periods reduce the synthesis of antioxidant molecules and enzymes. As a response to salinity, stomata close and photosynthetic activity decreases [9]. Molecularly, cell division could be regulated with reversible DNA damage or repaired DNAs, excess ROS could be removed, and metabolic homeostasis could be re-established with the help of exogenous treatments [7,48]. CRISPR and other molecular techniques could help with the development of new varieties and crops could be obtained, although stress symptoms are prevalent. The red color indicates antagonistic relations between the metabolites [49].

## 4. Improvement of Crop Plants under Saline Stress

Several strategies have been proposed for improving the conditions of plants under salt stress. Although the most efficient approach is to use resistant or tolerant cultivars, this is not always feasible. Alternative strategies should be used to lessen stress when the intended outcome is not achievable due to time, money, or the low tolerance level of plants. For example, Fan et al. reported that externally applied sodium nitroprusside (SNP), as a nitric oxide donor, reduced the effects of cold stress on Chinese cabbage seedlings when applied to leaves by increasing the antioxidant enzyme activities and protein contents of leaves while decreasing the stress levels (malondialdehyde, MDA) and membrane permeability [50]. Again, when 0.18 µmol L^−1^ GR24 was applied to *Brassica napus* L. at 0, 100, and 200 mmol L^−1^ NaCl doses, the deleterious effects of salt stress on root and stem growth, photosynthetic parameters, and leaf chlorophyll contents were reduced, the activities of POX and SOD enzymes increased, and MDA levels decreased [51]. Kausar et al. eliminated the negative effects of salt (150 mmol L^−1^ NaCl) stress in wheat by applying nitric oxide (0.05, 0.1 and 0.15 mmol L^−1^ SNP) and increased the antioxidant enzyme activities (POD, CAT, and SOD), total soluble protein accumulation and proline contents [52]. Abu-Qaoud et al. showed that a mixture of stress-tolerant bacteria and fungi could be effectively used under saline conditions [53]. They stated that they were able to grow wheat seedlings at a concentration of 6 dS m^−1^ NaCl, and the soil EC value decreased from 14.35 to 10.29 with the application of microorganisms at a concentration of 200 mLL^−1^ into the soil. Meena et al. showed that wheat grown under saline conditions (10 dS m^−1^) showed 92% germination success and improved biochemical and physiological parameters because of soil inoculation with halotolerant *Nocardioides* sp. and seed coating with bacterial extract [54]. They reported an increase in the expression of genes associated with tolerance. The survival, mycelial development, and sporulation of the bacterium under 10% NaCl conditions showed that the bacterium was adapted to high salinity conditions. *Nocardioides* sp. not only contributed to the growth and development of the plant under saline conditions by producing IAA in a culture medium containing 10% NaCl but also contributed to the defense by producing metabolites and significantly contributed to the reduction of stress levels in the plant. With this approach, although similar studies have been conducted before, we could reduce at least one of the impacts of stress factors. Alzahrani et al. stated that priming helped wheat plants adapt to 250 mmol L^−1^ NaCl [55]. Salt-pretreated plants synthesized lower MDA and exhibited saline-related gene expression (*TaNHX1*, *TaSOS1*, *TaSOS4*, *TaHKT1*, *TaHKT2*, and *TaAKT1*). Again, Saddiq et al. cultivated seeds of salt-tolerant (Kharchia 65) and salt-sensitive (PI.94341) genotypes of wheat plants after halopriming with 50 mmol L^−1^ KCl or NaCl or hydropriming with H_2_O at a 20 dS m^−1^ NaCl concentration until the formation of the fourth leaf and succeeded in reducing the negative effects of salt stress [47]. They reported that the priming effect at the germination stage contributed to DNA repair, respiration, transcription, translation, and the rapid functioning of energy metabolism. Similarly, Mohsin et al. showed that the effects of stress can be reduced by applying low doses of the herbicide hormone (2,4-D) to plants exposed to salt stress (150 and 250 mmol L^−1^ NaCl) [56]. They found that doses as low as 10 μmol L^−1^ of 2,4-D regulated the antioxidant enzyme and glutathione levels and plant nutrient uptake and reduced oxidative stress in wheat.

As seen from the recent findings, salt stress could be remediated by removing salt from the environment or encouraging the growth of plants under salt stress. The important point here is to evaluate whether this will work under combined stress, or do we need new approaches for the remediation of crop plants? Here, we list some recent works aiming to remediate saline soils or to improve plants under saline stress conditions in Table 1.

## 5. Responses of Wheat Plants Infected with *Z. tritici*

Before we evaluate the effects of the combined stress of pathogens and salinity, we will now discuss the pathogenic diseases of wheat plants. There are significant plant pathogens in wheat plants that reduce the quality and quantity. These are *Tilletia foetida* ve *T. caries*, *Ustilago nuda*, *Puccinia striiformis*, *Zymoseptoria tritici*, *Pseudocercosporella herpotrichoides, Drechslera sorokiniana, Fusarium* spp., *Rhizoctonia* spp., and *Sclerotium* spp. *Zymoseptoria tritici*, the causal agent of leaf blotch, is considered to be one of the most significant wheat pathogens. Although the infection and spread of the fungal agent are characterized by humid and cold conditions, the fungal agent has recently been observed in dry climate conditions characterized by salinity [80,81]. We noticed that newly developed wheat cultivars have thin wax layers that absorb light and facilitate efficient photosynthesis. Therefore, it is highly likely to develop interactions with other abiotic stresses. For example, Morgounov et al. reported that minimum and maximum air temperatures showed an increasing trend during the wheat growing season (April–August) from 1981 to 2015 in Canada, the USA, Russia, and Kazakhstan, and there was no declining trend in the appearance of *Z. tritici* infection in wheat-cultivated areas [82]. Therefore, the potential interactions between *Z. tritici* and various environmental stressors should not be underestimated. This fungus, which is widespread worldwide, has emerged as a particularly menacing foliar disease in the European Union, posing a threat to other regions due to global climate changes [83,84]. The fungus belongs to the Mycosphaerellaceae family of Ascomycota, and its sexual stage is known as *Mycosphaerella graminicola*. Morphologically, *Zymoseptoria* species are characterized by yeast-like growth in culture and the formation of various conidial types [85]. However, light conditions can influence the vegetative morphological structure of *Z. tritici*, and mutations in the *ZtvelB* gene can prevent the formation of yeast-like cells and asexual sporulation [86]. However, the fungus was able to produce micropycnidiospores capable of infection even when the *ZtvelB* gene was disrupted. *Z. tritici* exhibits a high level of genetic variation because of its capacity for both sexual and asexual reproduction within a single season. It is responsible for significant yield losses by limiting the photosynthetic area of wheat.

Goodwin et al. reported that *Septoria tritici* reduced the wheat yield by 30–50%, whereas Sidhu et al. stated that *Z. tritici* was considered a threat to global food security [87,88]. Although many protective fungicides are recommended to control the disease, they are not very economical in practice. Solomon reported that 70% of the annual fungicide applications were made to control *Z. tritici* in Europe, but even frequent spraying carried out during intense disease seasons, even one month before the harvest, did not control the disease [87]. Despite the use of fungicides against *Z. tritici* and the recommendation of resistant varieties for wheat cultivation, there is an annual crop loss of 10% in Europe alone, with a monetary loss of more than USD 1.5 billion [89,90,91,92]. Hehir et al. also stated that fungicide resistance in *Z. tritici* isolates has been a major threat to wheat production in Europe due to variations in the time and period of fungicide application [93]. If the time, labor, and financial budget spent on fungicides are added to this loss, a further USD 1 billion must be added. Therefore, the use of resistant varieties along with cultural measures is the most effective method. Bartosiak et al. reported that *Z. tritici* has the potential to adapt to temperate climate zones; therefore, climatic factors should be taken into account to control the spread of the disease [94].

### 5.1. Symptoms and Life Cycle of Z. tritici

Pathogenic fungi, in general, penetrate the host in four stages: first, mycelia or spores adhere to the host surface, form an appressorium, and penetration pegs then penetrate the host, colonize, and move into other tissues. At this stage, depending on the virulence of the fungus, some metabolites and toxins are produced and regulated by transcription factors and metabolic regulation programs [95]. During the penetration stage, contact and adhesion to the host cell are of great importance. The penetration ability of fungi that do not adhere well to the plant surface can be low, irrespective of how the appressorium is developed and if high turgor pressure is present, even if the mycelial structure is highly melanized [96]. Therefore, the presence of moisture in the environment under arid or saline conditions may be one of the crucial reasons why fungi become so aggressive under these conditions. Indeed, many authors have found a positive correlation between melanin production and pathogenicity. Fungi lacking melanin are generally considered unsuccessful pathogens. For example, mutant races of *Magnaporthe oryzae* lacking the melanin-synthesis genes *ALB1, RSY1,* and *BUF1* lost pathogenicity because of the very low turgor pressure in their appressorium [97]. However, in some cases, successful infection has been observed without the production of melanin [98].

The vegetative growth stages of *Z. tritici* are divided into three categories: micropycnidiospores, macropycnidiospores, and unicellular structures. Macropycnidiospores are the most common cell type, with multicellular structures comprising 4–8 long cells. Macropycnidiospores germinate into thin hyphae via polar tip growth [99]. This morphogenic transition between the structures can be induced by nutrient deprivation and temperature increases [100]. Micropycnidiospores consist of cells 1–110 µm wide and 5–10 µm long. Macro- and micropycnidiospores are found in dormant pycnids and are dispersed during rainy and wet weather [101]. The fungus carries 21 chromosomes, and 8 of these chromosomes (smaller ones) are called “dispersal chromosomes”, which have been found to carry genes related to pathogenicity [88,102]. These chromosomes facilitated the adaptation of the fungus to environmental conditions [103].

**Figure 2:**
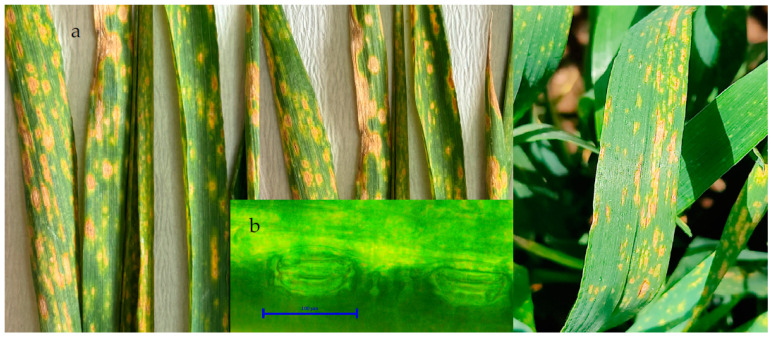
(**a**,**b**). Symptoms of *Z. tritici*: (**a**) characteristic symptoms of spots in leaf stripes and (**b**) penetration is performed mostly through stomata.

The first symptom of *Z. tritici* in wheat is the formation of chlorotic spots on the lower leaves close to the ground. At the initial stage, the symptoms are irregular, small with a light straw-yellow color in the center of the leaves that can be clearly distinguished. These spots progress and become irregular as the infection progresses, then turn into gray ashen and necrosis throughout the leaf. The small black dots on the spots are asexual pycnidia of the pathogen (Figure 2a). Pycnidia form in these lesions and gelatinous spores emerge from the pycnidia under moist conditions and are dispersed. Pycnids are usually brown, round, and 100–200 µm in diameter. Under favorable conditions, the pathogen penetrates through the stomata (Figure 2b). The pathogen overwinters as mycelium and pycnidium in plant debris.

### 5.2. Mechanisms of Disease Progression of Z. tritici

The primary infection starts via sexual airborne ascospores; rain and wind help the spread of ascospores [104]. *Z. tritici* usually infects its host through the stomata and very rarely penetrates directly through the leaves [105]. This pathogen causes major crop losses in areas where excessive fertilization and broad-leaved varieties are grown [106]. Secondary infections begin with asexual spores (conidia). Genetic recombination during sexual reproduction can produce more virulent pathotypes of *Z. tritici.* It is expected that the temperature should be favorable for the disease to start. Under high humidity, *Z. tritici* spores adhere to the leaf surface and germinate rapidly. Pathogenicity is initiated when the organism becomes dimorphic, i.e., from the spore to the hyphae. Following a dimorphic change, hyphae develop and grow with the aid of moisture, reach the stomata and penetrate it. The fungus colonizes the intercellular spaces in the leaves. Once inside, the hyphae disrupt the defense system by synthesizing effector proteins that break down the cell organelles. However, there is a debate about reaching the stomata. There is no clear evidence that hyphae penetrate the stomata by moving across the leaf surface. It is very difficult for a semi-biotrophic pathogen to utilize the organic matter on the leaf surface before becoming necrotrophic. It is still under debate how the fungus develops and moves to the stomata. If we could reduce the mechanism of infection, we could then evaluate the interactions of the fungus with other abiotic stress factors. For example, if we could extend the time of reaching the stomata and trigger the closure of the stomata, we would be highly successful in controlling *Z. tritici*. This could be an important step for the control of other leaf pathogens. Recent studies on this subject can be seen as a step forward to resolving these debates. Preliminary studies were conducted by Newey et al. [107]. They stated that *Stagonospora nodorum*, a major pathogen of wheat and other cereals, rapidly attached its conidia to hydrophobic leaf surfaces and secreted metabolites to ensure rapid and firm attachment. It is evident that when water droplets are present on the leaf, the wax layer and other hydrophobic biochemicals should prevent spores from adhering to the surface, thereby preventing further growth and development of the fungus. However, glycoprotein secretion facilitates the attachment of conidia to hydrophobic surfaces. Secreted metabolites, such as proteins and carbohydrates, are highly likely to break the hydrogen bonds that create surface tension in water. This may cause the rapid movement of the fungus and stomata. Although previous studies have reported that *Z. tritici* germinates on the leaf surface and moves toward the stomata, recent microscopic studies have shown that the mycelia move in the opposite direction to the stomata [105]. In very specific cases, hyphae growth toward the stomata was recorded. This supports our case for how the semi-biotrophic fungus quickly reaches the stomata. Rapid germination and movement of the conidia toward the vicinity of the stomata via this route require minimal energy. Hill and Solomon reported that *Z. tritici* hyphae slowly colonized the extracellular spaces of the mesophyll over a long latent period of approximately 8–11 days, depending on the pathogen isolate and wheat cultivar combination after the penetration stage [108]. During the latent period, the pathogen uses its lipid stores and fatty acids as energy sources. It is believed that effector proteins and small RNAs are prepared for mass production in the latent stage.

Pathogen transitions to the necrotrophic stage are characterized by pycnidia formation in the extracellular spaces, initiating secondary infections. At this stage, fungal biomass increases and all the conditions for colonization are met. This stage is also characterized by the suppression of host defense. During this stage, nutrients in the leaf cells are already released into the cell cavity to fuel fungal growth. This encourages the fungus to produce more pycnidia that disseminate pycnidiospores and spread infections to adjacent leaves and neighboring plants. This cycle is repeated many times during the development of wheat, and the pathogen can proliferate to the point of epidemics [92].

**Figure 3:**
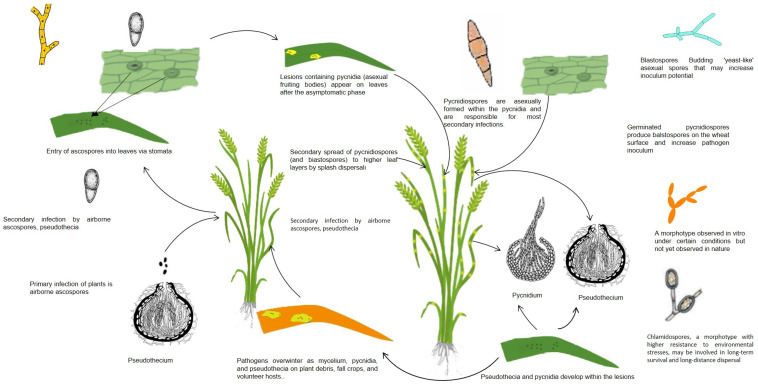
Life cycle and biology of *Zymoseptoria tritici*. Pycnidiospores emerge in spring from pycnids moistened by dew, irrigation water, and rain [111,128,129,130]. The spores infect the stems, leaves, and spikes of the plant, either directly or through the stomata. They then form pinhead-sized pycnids with necrotic spots and initiate secondary infections. Ascospores formed in the perithecia in late summer and autumn are dispersed in the air. They are deposited as pycnids or mycelium on seeds or infected plants in diseased plant debris.

*Z. tritici* differs from other leaf pathogens in terms of the infection process. *Z. tritici* encodes at least 24 carbohydrate-degrading enzymes [109]. Major changes were observed in the chloroplasts during the infection phase. The chloroplasts expand before collapsing, which is thought to be due to the synthesis of the enzyme laccase. *Z. tritici* produces small-secreted proteins (SSPs) that facilitate the completion of the infection process. When the pathogen moves into the cell from the apoplasts (extracellular spaces) to the symplasts (cytoplasm) system, signaling molecules produced by the pathogens’ avirulent genes are recognized by receptor proteins produced by the plants’ R genes, which characterize plant resistance. Once the signals are recognized, plants activate numerous defense mechanisms, synthesizing proteins involved in the construction of proline-rich cell walls and ROS, and activating genes and gene systems to synthesize resistance-related PR proteins such as SA and JA. Unlike other pathogens, *Z. tritici* does not form a specific structure but colonizes the apoplast spaces and absorbs carbohydrates dissolved in the apoplasts. If the host defense is adequate at this stage, further infection might be prevented; however, any additional stress factors, such as drought, salinity, or high temperatures, that are tolerated by the pathogen will decrease the defense responses and suppress the host defense and increase the pathogenicity, even if the pathogen is not virulent. A nutrient-limited environment may also act as a stimulus to induce a switch to an expletory structure as hyphae to search the environment for energy [108]. Therefore, the status of other stresses (salinity, drought, heavy metals, and cold stress) might play significant roles, and this has not been well elucidated. Although temperature has been suggested as the main reason for the transition to a dimorphic structure, i.e., an increase in temperature from 18 °C to 25–28 °C is effective in transitioning spores to a mycelial structure, this alone may not be sufficient. Indeed, the dimorphic structure is not immediately observed in wheat fields exposed to heat stress in the summer in many parts of the world. For example, Schwarczinger et al. reported that elevated temperature and powdery mildew (*Blumeria graminis* f.sp. *hordei*, *Bgh*) stress on resistant and susceptible barley plants showed that heat stress (24, 48, 120 h) enhanced the *Bgh* susceptibility in a susceptible barley line (MvHV118-17), whereas a resistant line (MvHV07-17) retained its pathogen resistance. [110]. However, prolonged heat stress significantly repressed the expression of several defense-related genes in both resistant and susceptibility barley lines. Switching to hyphal growth in dimorphic fungi through frequent anastomosis (two different mycelia share the same nucleus and cytoplasm) was considered to use the nutrient more efficiently in the mycelia under stress conditions. Francisco et al. reported that signaling systems in dimorphic fungi using hyphal fusions responded to stress very quickly [111]. The authors revealed that this also increased the virulence of the pathogens through easier colony establishment. The life cycle of the pathogen *Z. tritici* is illustrated in Figure 3.

While there is no clear difference between resistant and susceptible wheat varieties in terms of the pathogen penetration through the stomata, a clear difference is detected after penetration at the stage of disease progression. Therefore, controlling the disease with genetically resistant plants is the most effective method. Qin and LeBoldus showed that when poplar (*Populus* spp.) trees were inoculated with *Sphaerulina musiva*, the susceptible cultivar showed a late and weak defense response, and even if the entry point of the pathogen was thorough the stomata, the defense mechanism after penetration remained very weak compared with the resistant cultivar [112]. Similar results were also reported by Hemmati et al., who stated that soybean resistance was not important during pre-penetration, microsclerotia germination, and hyphae development of *Macrophomina phaseolina*; however, the post-penetration stage made a big difference between resistant and susceptible cultivars [113]. To date, the mapping of 22 major *Stb* (*Septoria tritici* blotch) genes and the discovery of more than 167 quantitative trait loci (QTLs) in chromosomes have been revealed in resistance studies [114]. Although *Stb* genes confer important resistance traits, virulent isolates may impair their stability [115]. The relationship between *Z. tritici* and resistant wheat cultivars is yet to be evaluated. For example, how and where to stop the pathogen in plant cells is of great importance. Indeed, the fungal pathogen can be arrested immediately after penetration of the stomata in both resistant and susceptible cultivars [116]. Many studies have reported the formation of hypersensitive responses and the accumulation of ROS and callose [117]. Proteomics and metabolomics studies have shown that early activation of carbohydrate metabolism, cell wall thickening, production of defense proteins, and production of antifungal metabolites in the apoplastic environment are effective [118] The basis of *Stb*-mediated resistance through the *Stb6* and *Stb16q* genes can detect fungal invasion in advance and activate defense signals [119,120]. Similarly, Battache et al. reported that a great proportion of hyphae penetrated the host largely because of the resistance conferred by the *Stb16q* gene [90]. Similar findings were also observed by Motteram et al., who reported that inactivation of the *ZtALG2* gene of the pathogen disrupted the dimorphic structure and prevented the infection of wheat [100].

Recent studies have determined the function of proteins associated with the pathogenicity of *Z. tritici*. These can be listed as transcription factors, kinases, signaling molecules, and cyclins [121]. Furthermore, deletion of the *ZtWor1, ZtVf1,* and *ZtRlm1* genes has been reported to inhibit developmental functions, asexual structure formation, virulence, hyphal branching, and dimorphic transition [122,123]. Furthermore, deletion of the *Zt107320* gene reduced the virulence of *Z. tritici* and reduced the infection in wheat [121].

To evaluate the penetration stage of *Z. tritici*, virulence-related genes should be revealed in detail. For example, Zhang et al. reported that the *ChCTR1* and *ChCTR4* genes of *Cochliobolus heterostrophus*, the causal agent of maize leaf blight, played significant roles in virulence [124]. However, mutant races lacking these genes involved in appressorium formation had low virulence. In particular, they found that copper ions were effective in appressorium formation and virulence. Therefore, it is necessary to know whether other ions, such as Na^+^ Cl^–^, etc., have a connection with existing virulence genes of *Z. tritici*. We need to evaluate the virulence genes under combined stress conditions. Any stress factors that result in the degradation of high-molecular-weight carbohydrates and proteins would further result in susceptibility to pathogen attack, since they become rich and ready to take in substrates for the development of the pathogens. Kou et al. reported that ROS produced upon stress in rice plants triggered the appressorium formation of the pathogen [125]. Similarly, Choquer et al. reported that the upregulation of sugar and carbohydrate degradation triggered protease and chitinase enzymes of *Botrytis cinerae* [126]. Therefore, sugars and low-molecular-weight sugar derivatives could be important substrates for pathogens.

Difficulties in developing resistant varieties at the desired level led researchers to search for alternative methods. The most effective, environmentally friendly method for the control of *Z. tritici* should deal with the process of inhibiting the conidial germination of the pathogen on the leaf. However, it remains unclear whether this can be achieved. A study revealed that a 50% reduction in sporulation was achieved either with MgSO_4_ or with a protein-based resistance builder (NECTAR Cereales) in wheat leaves [127]. The authors reported that the *POX2, PAL, PR1,* and *GLUC* genes were upregulated in the most susceptible cultivar (Alixan), whereas the *PAL* and *CHS* genes were activated by the phenylpropanoid pathway and upregulated in the most resistant cultivar (Altigo). From these findings, it is not possible to determine correlations between gene expression and resistance. Higher gene expression was found in susceptible plants, whereas low gene expression was found in more resistant plants [127]. These results demonstrate the complexity and specificity of the proposed defense mechanism.

Although the effect of NaCl on pathogens will be discussed in the next section, the same workers used salts to suppress pathogens. For example, El-Gamal et al. reported that silicate salts, potassium silicate, and sodium silicate were effective in suppressing *Z. tritici* and provided more effective results than chitosan, trisodium phosphate, glutathione, and fungicide applications [131]. Another study reported that the green seaweed *Ulva fasciata* induced resistance and provided protection to *Z. tritici.* Ulvan had no direct antifungal effect but reduced the fungal colonization and pycnidium formation in the substomatal spaces [132]. The authors reported that important genes related to phenylpropanoids (PAL and chalcone synthesis), oxalate oxidase, and lipoxygenase formation were not upregulated; metabolites did not show major changes, but minor metabolic changes were recorded, showing that wheat plants can be protected against *Z. tritici* without causing major metabolic changes. An important result of this study is that it brings a new understanding to breeding studies. Plants can be protected against disease without implementing major changes or genetic modifications. In particular, the expression of low-molecular-weight substances could play an important role in resistance without leading to major gene upregulation. This approach could be considered an important step for breeding studies, especially for plants under complex disease interactions. Indeed, the activation of low-molecular-weight metabolites through minor gene expression offers great advantages in terms of time, budget, and success. For sustainable resistance, the role of molecules or chemicals that continuously activate resistance is of great importance. For example, Mejri et al. stated that the application of saccharin (15 mmol L^−1^) to seedlings (wheat cultivar Alixan) 2 days before *Z. tritici* inoculation reduced the disease severity by 77%, although saccharin application did not show direct antifungal activity against spore germination and mycelial growth [133]. The authors reported that lipoxygenase (*LOX*) and pathogen-related *PR1* genes were upregulated after application; however, the *PAL* gene did not undergo a significant change, indicating that the disease severity can be reduced without altering key defense genes. Thus, triggering defense mechanisms could be achieved without major gene expression.

Biocontrol has also been successful in controlling septoria leaf spots. Allioui et al. found that bacterial protease and β-glucanase enzymes from *Bacillus subtilis* and *B. simplex* bacteria (Alg.24B1 and Alg.24B2) played an important role in pathogen control [134].

### 5.3. Role of Toxins and Pathogenic Enzymes of Z. tritici

*Z. tritici* causes significant damage to the host with toxins or similar microproteins. Liu et al. found that the toxin obtained from *Stagonospora nodorum*, SnTox1, was a host-specific toxin and its activity only decreased at 50 °C in in vitro conditions [135]. This clearly showed that toxin-producing fungi cannot be easily controlled by temperature or other methods. Recent studies on *Z. tritici* have noted the presence of toxin and toxin-synthesizing genes. Mirzadi et al. detected *TOX2* genes in *Cochliobolus carbonum* and showed that the same gene group was present in *Z. tritici* [136].

*Z. tritici*, following penetration, activates genes encoding lysine-containing proteins (*LysM, Mg1LysM, Mg3LysM*, and *MgxLysM*). The first two of these proteins increased during the asymptomatic stage of *Z. tritici* infection and attached to chitin in the leaves. The Mg3LysM protein plays a vital role in the host immune system. These proteins play significant roles in the biotrophic phases that determine whether and how the infection continues. In the necrotrophic stage, transcription of virulence genes is a crucial step for the onset of further infections.

The fungi use a range of weapons to emphasize their aggressiveness. These can be considered physical or chemical. Chemically, fungal virulence is characterized by enzymes that degrade the host cell wall, metabolic processes such as carbohydrate and protein metabolism, and toxins that impair respiration and photosynthesis. Physically, the fungus uses appressorium and penetration pegs to invade the cell wall. Any organic compounds in the vicinity of the fungus might be used as energy sources. If these compounds have low molecular weights due to the breakdown of macromolecules because of previous stress, chemical and physical weapons could be used efficiently. In particular, amino acids, low-molecular-weight carbohydrates, i.e., sugars and sugar derivatives, minerals, and ions (Mg^2+,^ Mn^2+^, Na^+^, etc.) are thought to contribute to the turgor pressure. For example, Dikilitas et al. reported that proteases and glucose molecules were effective for the virulence of the fungi *Pyrenophora teres* f. *maculata* (*Ptm*) and *Pyrenophora teres* f. *teres* (*Ptt*), the causal agents of spot and blight diseases in barley [6]. They found that the more virulent *Ptm* isolates synthesized more protease and a higher glucose content than the less virulent *Ptt*. We noticed that the fungus can produce more of these enzymes in susceptible plants. For example, the protease activity of *Fusarium oxysporum* f.sp. *dianthi* was compared in resistant and susceptible carnation (*Dianthus caryophyllus* L.) plants, and the fungus synthesized higher levels of protease in susceptible plants [137]. Therefore, any additional stress factors in plants that would reduce tolerance or resistance levels might encourage the synthesis of protease or laccase enzymes.

Protease and laccase enzymes have important effects on the metabolism, physiology, and development of fungi. Many studies have shown that proteins, known as effector proteins, are synthesized by fungi in the host cell, which could lead to modification of the host protein. In particular, a strong positive correlation was established between the protease enzyme levels and disease progression in plants [138]. Han et al. reported a positive correlation between the protease enzyme levels and the virulence of the fungus *Scedosporium aurantiacum* [139]. Ben Ali et al. found that *Trichoderma asperellum* produced high levels of the enzyme laccase in 18 mmol L^−1^ CuSO4 or 1% NaCl, a salt concentration close to seawater levels [140]. Fungi that tolerate salt stress can easily produce pathogenic enzymes. For example, Damare et al. reported that heavy metal ions (Cu, Hg, Fe, and Ni) did not inhibit the fungal enzyme of *Aspergillus ustus* that adapted to cold conditions. The activity of the fungus was maintained at 2 °C. The fungus could produce protease enzyme even in 0.5 mol L^−1^ NaCl medium [141]. Therefore, adaptive characteristics in relation to abiotic stress factors could create cross-tolerance to other abiotic stress factors in fungi.

Once a fungus can produce protease and laccase enzymes, it is very difficult to reduce the level of these enzymes by modifying the habitat temperature of the fungus. For example, Kılıç et al. demonstrated that the fungus *Penicillium expansum*, a causal agent of decay in apple and tomato fruits, produced protease and laccase enzymes both at 4–8 °C and at 35 °C and above [80]. The release of glucose was also determined followed by the inoculation of *Septoria tritici* in wheat [142]. It was observed that the pathogen broke down the macromolecules into smaller ones. The role of protease and other enzymes was also revealed in *Z. tritici*. Amezrou et al. reported that protease secreted by *Z. tritici* hastened the adaptation process [143]. The synthesis of cell-wall-degrading and protease enzymes was regulated differently according to the resistance levels of the host plants. For example, Somai-Jemmali et al. reported that spores of *Z. tritici* easily germinated and penetrated the susceptible host since neither the defense mechanism through PR-protein-encoding genes nor the pathogen was recognized at earlier stages [144]. On the other hand, the pathogen gained entrance through stomatal penetration in resistant cultivars. The authors stated that during direct penetration, cell-wall-degrading enzymes and protease enzyme activities played significant roles. The level of enzymes such as xylanase, protease, and cellulase, were correlated with the disease severity and this was used to assess the range of susceptibility of wheat cultivars to *Septoria tritici* infection [145]. Although *Z. tritici* does not penetrate plant cells at any stage of infection and has a long latent period of symptomless colonization on leaf surface [146], several important primary and secondary metabolites after abiotic stress occurrences changed the pathway of pathogenicity. So, any reductions in defense barriers due to the involvement of abiotic stress factors such as drought and salinity and the elevation of the pathogenic enzymes would encourage the direct penetration of *Z. tritici* through the leaf epidermis.

Melanin also plays a crucial role in the survival of fungi under stress conditions. Freitas et al. reported that melanin production was directly related to pathogenicity and virulence in many fungi [147]. Similarly, Aranda et al. reported that melanin in darkly pigmented *Gaeumannomyces graminis* var. *tritici (Ggt)* triggered pathogenicity in wheat plants [148]. Fungal melanins not only neutralize ROS and other radical ions produced by the host but also play important roles in conidial adhesion to the host cell wall, as shown in *Aspergillus fumigatus* [149]. Therefore, any fungi that can produce melanin have a great capacity to escape from antagonistic microorganisms and protect themselves from biotic and abiotic stresses. Tilet et al. established a strong relationship between melanization and virulence in *Z. tritici* [150]. Similar issues were also raised by Kılınç et al., who stated that melanin accumulation in *Z. tritici* increased up to 30 °C in terms of air temperature. The authors stated that melanin increases might be one of the reasons for ongoing pathogenicity. Given the accumulation of metabolites and phenolic compounds in plants after exposure to abiotic stresses, it is not difficult to predict that the fungi using metabolites and phenolic polymer compounds as substrates would either be more pathogenic or would carry out their pathogenicity. Therefore, any metabolite accumulations followed by stress in plants could be used by the attacking pathogens, with no exceptions for leaf or root pathogens.

## 6. Responses of Cereal Plants and Pathogens to Salinity Stress

### 6.1. Adaptation of Fungi to Stress Conditions

Microorganisms, particularly fungi, can adapt to the environment in which they thrive. These environments can be heavy metal-, salinity-, or pesticide-contaminated or high temperature-, cold-, or drought-characterized areas. Fungi can adapt to harsh environments by producing high levels of amino acids (proline, glycine betaine), and sugars such as glucose, trehalose, or microproteins [151]. For example, Dikilitas found that salt-tolerant isolates (150 and 200 mmol L^−1^ NaCl tolerant) of *Verticillium albo-atrum* developed faster than salt-intolerant isolates under 50, 100, 150, 200, and 250 mmol L^−1^ NaCl conditions, producing enough conidia to start the infection [7]. The authors reported that the isolates were capable of infecting tomato and lucerne plants under normal and saline conditions. Similar results were obtained by Hagiwara et al. concerning *Aspergillus fumigatus*, which tolerated 37 and 45 °C [152]. The fungus produced trehalose and melanin, and it upregulated dihydroxynpthalene (DHN)-melanin-related genes at high temperatures. Similarly, Liu et al. showed that the mycelial growth of *Setosphaeria turcica* (the leaf blight pathogen of maize, sorghum, and many Gramineae) was reduced but the metabolite efflux was greatly accelerated in 400 mmol L^−1^ NaCl, 400 mmol L^−1^ KCl, and 600 mmol L^−1^ sorbitol conditions [8]. All salt-tolerant wheat species cannot perform well over 250 mmol L^−1^ NaCl doses; however, as indicated in the previous sections, pathogens, in general, adequately sporulate and mycelial growth carries on although the reduction in these growth parameters is reduced [153]. Some researchers suggested that the application of salt compounds could control septoria leaf blotch infection [131]; however, fungi cannot be controlled by alternative chemicals to fungicides. Fungi could likely develop tolerance to those chemicals as well. Previous studies have shown that microorganisms exposed to increasing levels of abiotic stress develop tolerance to one stress factor while developing tolerance to other stress factors. This is known as cross-protection or cross-resistance and the mechanisms discovered in other organisms were also found in fungal cells. In this mechanism, the previous stress remembered by the fungi helped quicken adaptation to subsequent stress conditions than fungi that had never been exposed to stress before [154,155]. Dikilitas et al. reported that *Verticillium dahliae* and salt-adapted *V. albo-atrum* were able to produce sufficient conidia and mycelia without losing their pathogenicity and carried on infections in tomato plants [6,7]. Apart from the production of metabolites, such as glucose, trehalose, glycerol, etc., under saline conditions, a vesicular structure such as rhamnolipid was found to be directly linked with the aggressiveness of *Pseudomonas aeruginosa* [137]. A giant rhamnolipid structure that accumulates ca. 375–875 mmol L^−1^ NaCl in *P. aeruginosa* could be observed in other pathogens, and more virulent races of the fungi could appear.

Not only can fungi survive and adapt to saline stress conditions by synthesizing metabolites but they are also stimulated and produce conidia and mycelia profoundly and increase the mobility of conidia. Boumaaza et al. reported that conidial production and germination of *Botrytis cinerea* were stimulated by salt [156]. The authors suggested that growers should not irrigate crop plants with saline water, even if the irrigation water contains a low amount of salt. Even if the fungi lose the ability of conidial germination and show slow development, they can still adapt to saline conditions over time. This type of fungus can be dangerous even in non-saline conditions [157,158]. Kılınç et al. studied four different isolates of *Z. tritici* under different temperature conditions (4, 15, 25, 30 and, 35 °C) and found that the number of spores per unit volume (mL) and the average growth diameter decreased, together with the spore density per unit area (cm^2^), as the temperature moved away from the optimum value (20 °C); however, the synthesis of protease and laccase enzymes and conidial production were sufficient to carry out the infection process [159].

**Figure 4:**
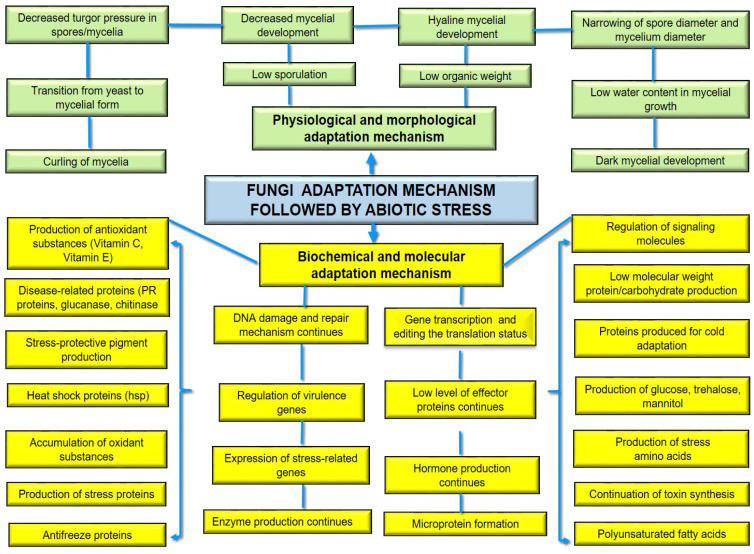
Adaptation mechanisms of fungi after stress. If fungi can produce the metabolites illustrated in the above diagram, then they can easily adapt to harsh conditions by reducing the metabolites and substances, such as spores, mycelial development, and biochemical compounds, normally produced under stress-free conditions. Molecular adaptation mechanisms under abiotic stress conditions are differently regulated [5,108,161,162,163,164]. It is important to point out here that metabolites produced under normal conditions are not nullified, they are minimized, and some extra metabolites are produced to cope with the stress [7,137,151,154,165,166].

The adaptation mechanisms of the fungi are illustrated in Figure 4 under the scope of general stress factors. If we look at this adaptation and pathogenicity mechanism from the fungal point of view, pathogenic fungi increase the signaling pathways, transcription factors, and gene expression levels while maintaining their pathogenicity with the proteins and toxins they synthesize [31,95,160]. From the point of view of the host plant, the signaling systems that lead to plant immunity and the regulation of the structural, physiological, and biochemical responses of the plant have been identified [22,40,111,154]. If the behavior of wheat pathogens and the mechanisms of wheat plants under salt stress are well understood, it is possible to breed both tolerant and resistant wheat plants for the combined stress. The state of the art of agriculture should consider the combined stress issues due to global warming and environmental pollution.

### 6.2. Responses of Cereal Plants to Saline and Pathogenic Stress Conditions

We have mainly evaluated salinity and pathogen stresses in cereal plants in separate sections, each of which poses significant threats to plants. Although salinity and pathogen combinations exert stress on crop plants, salinity also causes stress in pathogens. When they are combined, either in simultaneous or consecutive combinations, they have drastic consequences for plants. The important issue here is the tolerance level of pathogens under combined stress. For example, McCorison and Goodwin showed that more than 3400 genes were upregulated in *Z. tritici* grown under white, blue, and red light for 1 h compared with the fungus grown in the dark. They showed that effector proteins upregulated under light conditions played a stimulating role in pathogenicity [167]. Similar attributions could be made to other stress factors for the pathogenicity of *Z. tritici*.

Despite advancements in the development of resistant crop varieties against pathogens, the breakdown of resistance barriers and pathogen adaptation to existing abiotic stress conditions hamper breeding efforts in the field and complicate future studies. It is crucial to elucidate the defense mechanisms of wheat plants under combined stress conditions. In addition, it is essential to understand the role of genetic factors involved in defense mechanisms. Failure to do so may impede the desired success in breeding and disease control. A detailed understanding of the mechanisms under dual stress conditions will guide breeding studies and enhance the production per unit area for sustainable crop production strategies. Combined stress conditions can be simulated through in vitro, field, and greenhouse experiments, and this knowledge can be used to generate disease-resistant or stress-tolerant cultivars.

In regions characterized by frequent rainfall and cooler climates, crop yield losses caused by wheat pathogens can be more pronounced, reaching 40%. These diseases are primarily caused by airborne pathogens and can rapidly and extensively infect their host plants. Unlike soil-borne fungal pathogens, they pose a more immediate and severe threat due to the rapid and intensive spread of their inoculum. In recent years, it has become evident that pathogens interact with various abiotic stress factors, and some environmental conditions can intensify the proliferation and spread of pathogens, as demonstrated by Dikilitas et al. [5]. Before we move on to the combined effect of salinity and disease stress on crop plants, we will list the general growth conditions of plants and pathogens in saline conditions in Table 2.

The interaction between pathogens and salinity stresses is not limited to plant and soil-borne pathogens but also extends to leaf-infecting pathogens. However, when abiotic stress conditions affect foliar pathogens, the consequences are expected to be more severe. This may be due to the faster and more effective spread of airborne pathogens and their higher sporulation rates. Consequently, it is predicted that the molecular and biochemical adaptation mechanisms of these pathogens will become more advanced, considering that leaf-borne pathogens are likely to encounter a higher number of environmental stress factors.

**Figure 5:**
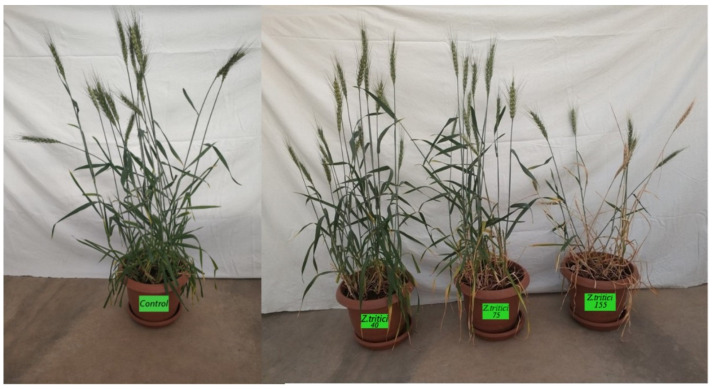
Wheat plants inoculated with *Z. tritici* under 40, 75, and 155 mmol L^−1^ NaCl conditions compared to the control plants (pot on the left side). Increased NaCl concentrations have devastating effects. Plants inoculated with only *Z. tritici* or treated with low concentrations of NaCl might recover from the negative effects of the pathogen or NaCl or the effects of combined stress if the NaCl concentration is low. However, high doses of NaCl may not allow recovery. The photos are of the original work of the Ph.D. study by the first author.

Combined stresses involving both a pathogen and salt stress may occur either sequentially or simultaneously. In such cases, the defense mechanisms of plants can be affected in unpredictable ways. Because abiotic and biotic stresses activate different signaling molecules and enzymatic activities in plants, these signaling molecules can interact negatively and disrupt the defense system at an early stage, as shown in Figure 5. In plant breeding, the development of plants capable of resisting both stress factors will be credited. In other words, no matter how resistant a plant is to the attaching pathogen, its defense mechanisms are impaired when exposed to abiotic stress factors [5,6].

One of the main challenges in developing resistant and tolerant crop plants is the complex mechanisms of combined stress. We must consider two different stress factors that do not share common pathways. For instance, plants may be exposed to abiotic stresses (temperature, drought, high light intensity, frost or chilling injury, water stress, heavy metals, salt stress, etc.) and biotic stresses (bacteria, viruses, fungi, phytoplasma, insects) either simultaneously or sequentially. Although combined stressors have not been considered very much in the past, it is now possible to face both types of stress factors in nature. Generally, when a plant is exposed to biotic stress, it activates its defense mechanisms, while in the presence of abiotic stress, it tries to tolerate or escape from the stressors by storing ions if involved or synthesizing compounds to tackle the stress [6]. This can involve actions such as stomatal closure or an increase in respiratory capacity to produce energy, depending on the situation. Even if plants are genetically resistant to such conditions, chronic stress can disrupt structural and biochemical mechanisms. In most cases, when plants are exposed to both types of stress factors, a combined effect or additive effect of both stress factors is observed, causing the defense system to deteriorate rapidly, as in the equation of “2 + 2 = 4” [5,179]. In some cases, this scenario can be even worse, such as the equation of “2 + 2 = 8”, where the abiotic stress factor stimulates the growth and proliferation of the pathogen by increasing the pathogenic enzymes and toxins [6]. In very rare instances, one of the stressors may not be observed, as described in the equation of “2 + 2 = 2”. This typically happens when the biotic stress factor remains dormant due to the dominance of the abiotic stress factor. Conversely, as indicated by the equation of “2 + 2 = 1”, some plants infected with pathogens might show greater tolerance to drought or temperature. It has been revealed that plants may synthesize drought- or temperature-related proteins, and these molecules could be used to stop pathogenic infections or vice versa [46,180]. In most cases, scenarios 1 and 2 are the most commonly observed in nature. In recent years, scenario 2 has been on the rise [181,182]. One of the key reasons could be the firm establishment of abiotic stressors over time, allowing adaptations of pathogens to abiotic stress conditions.

Regardless of whether abiotic and biotic stresses affect plants simultaneously or sequentially, the mechanisms by which these two types of stresses affect plants have not been extensively highlighted. Under abiotic stress factors, plants produce glucose based on the production of six-carbon sugars to cope with stress issues and regulate osmotic pressure, toxicity, mineral nutrient imbalances, and water uptake [183]. On the other hand, under biotic stress, plants typically produce five-carbon sugars using the pentose pathway, along with increased respiration, secondary metabolite formation, and structural defense systems such as suberin, lignin, callose, cellulose formation, increased turgor pressure, and other structural changes. The negative impact on photosynthesis is also reflected in the crop yield [184]. In some cases, biotic stresses can directly affect photosynthesis by impacting the mobilization of crucial elements such as Mg^2+^ and Mn^2+^ through the toxins and microproteins they produce [165]. Direct targeting of photosynthesis can also occur under high concentrations of heavy metals or salts [185]. For example, under high NaCl concentrations, Na^+^ ions entering the cell can directly affect photosynthesis by replacing K^+^ ions in the stomata, keeping them continuously open, leading to increased water loss and reduced photosynthesis [186].

In some cases, the defense mechanisms of plants can drastically deteriorate and cannot be repaired unless a stressor is removed. For example, Dikilitas et al. found that the total antioxidant metabolites of tomato plants inoculated with *Verticillium dahliae* and irrigated with 50, 100, 200 and 250 mmol L^−1^ NaCl significantly decreased, whereas the oxidative stress metabolites increased [171]. Similar issues were reported by Al-Hammouri et al. [187].

Another significant aspect is that in many combined stress studies, abiotic and biotic stresses act on the same conductance bundles, such as xylem bundles. When different types of stress agents travel through the same vessel element or conductance bundle, the severity of the stress could be different from those they occupy in different vessel elements (e.g., one stress agent could target phloem while the other stress agent could target xylem vessel elements) [188]. However, a detailed understanding of the defense and tolerance mechanisms, as well as the molecular and biochemical pathways, needs to be evaluated in detail. For instance, foliar pathogens such as *Zymoseptoria* spp., *Cercospora* spp., and *Erysiphe* spp., which infect through the phloem conduction bundle, may interact with abiotic stresses such as NaCl, which uses xylem vessel elements and may impair the photosynthesis metabolism rapidly. Although plants possess pre-existing physical, biochemical, and molecular barriers against abiotic or biotic stresses, these barriers might not be sufficient when the combined stress threatens crop plants. For example, plants exposed to drought stress might be vulnerable to disease infections. De Pascali et al. stated that the *Xylella fastidiosa (Xf*)-resistant cultivar Leccino subjected to individual drought or pathogen infection and to the combination of both exhibited pathogen-related *PR* and leucine-rich repeat genes, *LRR-RLK*, and these genes were not further induced by a water deficit in the combined stress [189]. They stated that genes can respond to simultaneous stress differently than additives. A similar scenario was found between cold stress and powdery mildew [190].

One of the negative effects of salt when combined with the pathogen is that low concentrations of salts could be easily tolerated by the fungi while inducing ABA signaling, which interferes with SA-mediated defense responses. This may result in increased susceptibility in plants [180]. However, pathogen stress also affects ABA signaling; therefore, pathogenicity under drought or saline conditions has negative effects under combined stress conditions.

**Figure 6:**
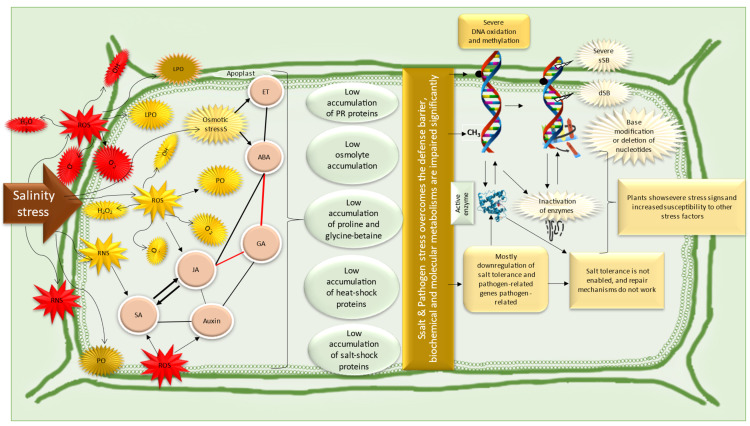
Overview of the combined effects of salinity and pathogen stress on cell metabolism. The red color indicates the antagonistic relations between metabolites. ROS, reactive oxygen species; RNS, reactive nitrogen species; ABA, abscisic acid; GA, gibberellic acid; SA, salicylic acid; JA, jasmonic acid; ET, ethylene; LPO, lipid peroxidation; PCD, programmed cell death; sSB, single strand breaks; dSB, double-strand breaks; PC, protein carbonylation; PO, protein oxidation. The illustrations and mechanisms shown here were prepared according to the works of [48,158,191,192,193].

We have summarized the interactions between pathogens and salinity, mainly highlighting the status of wheat plants, in Figure 6.

Under the combined stress of salinity and pathogen interactions, osmotic stress, ionic imbalance, toxicity of ions, and pathogenic enzymes and toxins severely disrupt plant defense mechanisms [57]. Excessive toxicity of Na^+^ and Cl^−^ ions causes an ion imbalance and leads to physiological drought, even if proper irrigation is performed. Wheat plants, even if they become moderately salt-tolerant or disease-resistant, show devastating metabolic changes, which are reflected in their morphological traits. The accumulation of salt shock and PR proteins in salt-acclimated or -adapted plants may show antifungal activity if the plant is moderately resistant to disease or tolerant to salinity. However, the accumulation of high levels of ROS impairs the cell defense system and results in membrane perforation and solute leakage from the cell, and communication inside the cell is disrupted or lost in severe cases [27]. Modification in fatty acid, protein, carbohydrate, and lipid compositions and increases in glucose and amino acid levels could act as a good source of substrates for pathogens. High sugar content and amino acids encourage the growth and sporulation of fungus if pathogen infection continues [6]. Regulation of genes is interrupted, DNA fragmentation, methylation, oxidation, increase in a single strand of DNA breaks, and in severe cases, double-strand of DNA breaks could occur. ABA results in the closure of the stomata, which prevents water loss and pathogen entry; however, overproduction of ABA results in the enhancement of pathogen spread and sporulation. Under salinity stress, ABA and ET have negative correlations with other hormones, such as SA, JA, GA, auxin, etc.; therefore, suppression of such hormones decreases defense mechanisms and causes susceptibility to pathogen attacks [63,194]. PCD may not work efficiently; therefore, sudden death is inevitable. Suppression of growth hormones and triggering of stress hormones increase the antioxidant enzymes at the first stage, then the sudden depletion of enzymes and metabolites leaves the plants undefended. Increased lignin and suberin syntheses could be observed, but the sudden decrease is evident; therefore, early senescence and defoliation are inevitable. Plants under multiple stress conditions cannot synthesize antimicrobial compounds. Thus, the progress of the invading fungus becomes very fast. The behavior of the stomata, whether they should remain open or closed, would affect the responses of wheat plants. In either case, wheat plants suffer from severe stress, e.g., if the stomata remain open, plants will lose water and cannot grow well and show signs of wilting and chlorosis along with other symptoms; or if stomata remain closed, the plant will preserve water but cannot produce enough proteins and other metabolites to function. Most importantly, defense proteins are not produced, and fungal attack through the stomata is very severe. Molecularly, cell division may not occur or the cell divides with irreversible DNA damage. Excess ROS cannot be removed, and metabolic homeostasis cannot be sustained. Exogenous treatments, such as antioxidant supplies or priming effects, may not solve this problem. Newly formed cells do not function properly and may not produce sufficient defense metabolites. Syntheses and functions of salt-shock proteins or PR-proteins may not be sufficient for both stress factors. Both resistant and tolerant varieties should be generated to obtain sufficient crop production. 

## 7. Mitigation Strategies for Salinity-Induced Pathogenicity in Cereal Plants Exposed to Saline and Pathogen Stressors

Because of multiple or combined stress conditions, we should employ a multigene system program for resistance in wheat studies. The resistance of wheat plants to the effects of secondary metabolites, effector proteins, and mycotoxins at the primary and secondary infection stages is quite complex and requires long-term studies. Clustered Regularly Interspaced Short Palindromic Repeats/CRISPR-based (CRISPR/Cas) systems could be used to mutate sensitive sites in the wheat genome to confer resistance to fungal diseases or salinity stress. The CRISPR/Cas system is a working system that deals with the reorganization of the genome region. Having identified the specific regions of DNA, we could develop new wheat varieties. We should understand the pathways of salinity and disease stress. We know that root pathogens, in general, use xylem vessel elements such as NaCl; however, leaf pathogens directly penetrate through the leaf stomata or wounds into subcellular cavities in the leaves and use mostly phloem translocation system, and they directly inhibit photosynthesis via inhibiting translocation of Mg^+^, Mn^2+^, Zn^2+^, Ca^2+^, K^+^, etc. [195]. How xylem-inhabiting pathogens differ from phloem-inhabiting pathogens, how root (soil-borne) pathogens differ from leaf-infecting pathogens, or their combinations with those associated with other abiotic stress factors should be well evaluated rather than reporting the effects of combined stress.

Any suggestions for crop improvement, such as biochemical, molecular, breeding, or nanotechnological approaches, should be well evaluated. It is important to reduce the effect of at least one of the stressors to grow wheat plants exposed to combined stress. The important issue here is to deal with the abiotic stress factor, as pathological agents could hide themselves or act more aggressively, as described in the previous section. We have summarized the physiological, biochemical, and molecular approaches for improving wheat plants under the combined stress of salinity and pathogen stress in Table 3.

Exogenous applications such as signaling molecules, microbial applications, or remediation of saline soils, etc., could not improve the conditions of plants alone; however, it is useful to nullify, neutralize, or reduce the impact of one stress factor, e.g., salinity. Gene modification (transgenic approach), OMICS applications (transcriptomics, proteomics, miRNAomics, metabolomics, etc.), and priming studies could help in the regeneration of tolerant and resistant wheat plants.

## 8. Future Directions and Conclusions

As our understanding increases in terms of the defense responses of plants to salt and pathogen stresses, we could generate more resistant and tolerant crop plants without touching the crucial parts of the crop plants, such as the quality and taste. By unraveling the molecular mechanisms of wheat plants under combined stress conditions, new wheat lines could be produced with less energy, time, and budget. By integrating multidisciplinary approaches and harnessing the power of genomics and other elements, we should act swiftly to develop new lines; otherwise, the loss of crops could be detrimental.

Since we could deal with the studies of metabolomics and proteomics intensively in recent years, we could obtain the results much quicker to come to a firmer conclusion than before. We should start by identifying the roles of metabolites and their expressions in abiotic, biotic, and abiotic and biotic stress conditions. We should identify the metabolites that behave antagonistically or synergistically in the networks so that any approaches to increase the defense metabolites could easily be detected if they interfere with other metabolites. Although the combined mechanism of pathogen and salinity interactions is very complex, we could at least tackle the case with little effort by modifying or regulating the behaviors of the stomata so that we could restrict the entry of the pathogens. For example, we can arrange the timing of fertilizer applications and possibly delay the interference of the leaf pathogens existing on the leaves with the ionic parts of inorganic compounds to restrict the mobility, sporulation, and mycelial development of the pathogens that might otherwise be increased with the low concentrations of Cl^−^, Na^+^, and other ions. We could also deal with minor gene expressions. Instead of working on a major gene expression or a group of genes using the single pathway to increase the defense mechanisms, multiple minor gene expressions using many alternate pathways should be evaluated if the desired defense success is achieved. Since this approach does not interfere with the quality parameters, one should consider this approach. Chemicals with priming effects or seed priming studies should be encouraged, at least to tackle one of the stress factors in advance. New signaling molecules that quickly evoke plant defense systems should be tested to see if multiple stresses can be prevented before they happen. New chemical compounds that are commercially available might have promising results before we generate genetically resistant or tolerant wheat plants for multiple stress factors.

## Figures and Tables

**Table 1:** Salinity stress in plants, particularly wheat, and their improvement via physiological, biochemical, and molecular mechanisms *.

Salinity Stress (Leaf/Root)	Characteristic Symptoms and Improvement Strategies	References
150 mmol L^−1^ NaCl + Drought (Root)	Salinity and water deprivation caused significant growth retardation and a 3-fold reduction in the osmotic potential in wheat seedlings. Polyvinylpyrrolidone (PVP)-coated Cu nanoparticles in *Triticum aestivum* under salt and/or drought stress restored the growth rate and regulated water uptake.	[57]
120 mmol L^−1^ NaCl, Root	Exogenous silicon nanoparticles (SiNPs) on *Elymus sibiricus* seeds increased the chlorophyll and osmolyte accumulation and improved the activities of SOD, CAT, POD, and APX.	[58]
Root (2% NaCl)	Exogenous sodium nitroprusside (SNP, a donor of NO) increased the lignin and salicylic acid (SA) contents under normal and salinity (2% NaCl) conditions via triggering the phenylpropanoid pathway as well as increasing the level of transcription of the pathogenesis-related protein 1 (*PR1*), *TaPAL* and *TaPRX* genes responsible for the syntheses of POD, PAL, and tyrosine ammonia lyase (TAL) enzymes.	[59]
Root (0–250 mmol L^−1^ NaCl)	Wild common wheat species were proposed for the improvement of wheat cultivars due to possessing resistance genes to various environmental stresses via synthesizing CAT, SOD, and APX and expressing their encoding genes.	[60]
Root (50–150 mmol L^−1^ NaCl)	Seed priming with NaCl was suggested to improve plant growth by altering the Na^+^ and K^+^ content under salt stress.	[61]
Root (150–200 mmol L^−1^ NaCl	The roles of the microRNA (miRNA) family in modulating plant growth and development through tae-miR9674a were proposed as an essential mediator in the wheat plant for the osmotic stress tolerance, cellular ROS homeostasis, and defensive processes.	[62]
Root (0–200 mmol L^−1^ NaCl)	Exogenous jasmonic acid (JA) significantly improved the salt tolerance of wheat seedlings via alleviating membrane lipid peroxidation and enhanced the contents of abscisic acid (ABA), jasmonic acid (JA), and salicylic acid (SA). In the RNA-seq profiles, 2% of the unigenes were differentially expressed upon application.	[63]
Root (0–250 mmol L^−1^ NaCl)	SA application enhanced the salt tolerance of perennial ryegrass (*Lolium perenne* L.) via improving photosynthesis, stomatal conductance, and defense systems.	[64]
Root (250 mmol L^−1^ NaCl)	Salt-pretreated plants maintained higher photosynthetic efficacy and lower proline and MDA contents than non-pretreated plants. Salt-pretreated plants also sustained high expressional levels of salt-responsive genes (*TaNHX1*, *TaSOS1*, *TaSOS4*, *TaHKT1*, *TaHKT2*, and *TaAKT1*).	[55]
Root (350 mmol L^−1^ NaCl)	Two neglected (*Aegilops triuncialis* and *Ae. tauschii*) wheat genotypes responded better to salinity stress, and they were suggested for breeding lines to improve salt tolerance in wheat plants.	[65]
Root (150–250 mmol L^−1^ NaCl)	*Triticum aestivum* L. cv. Norin 61 treated with 2,4-dichlorophenoxyacetic acid (2,4-D) herbicide increased the glyoxalase enzyme activity that enhanced salt tolerance via inducing methylglyoxal (MG) detoxification systems.	[56]
Root (0–200 mmol L^−1^ NaCl)	The salt-tolerant diploid (*Triticum monococcum* cv. Turkey) cultivar accumulated less toxic sodium in the photosynthetic tissues with the high expression of *SOS1*, *HKT,* and *NHX* genes in the leaf blade than the salt-sensitive tetraploid wheat (*T. durum* cv. Om Rabia3).	[66]
Leaf (0–100 mmol L^−1^ NaCl)	GR24, a synthesized strigolactone plant hormone, alleviated salinity tolerance in rapeseed (*Brassica napus* L.)	[67]
Root (150 mmol L^−1^ NaCl)	*Trichoderma longibrachiatum* T6 (T6) promoted wheat growth and induced plant resistance under saline conditions.	[68]
Leaf (0–100 mmol L^−1^ NaCl)	Foliarly applied 24-epibrassionlide (24-epiBL) alleviated the adverse effects of salt on wheat by enhancing the photosystem-II efficiency.	[69]
Root (EC = 10 dS m^−1^ NaCl)	The application of arbuscular mycorrhizal (*Glomus deserticola*) mitigated the reduction of K, P, and Ca uptake in sweet basil (*Osmium basilicum*) plants under high saline conditions.	[70]
Root (0–200 mmol L^−1^ NaCl)	The plant growth-promoting (PGP) bacterial isolate CDP-13 from *Serratia marcescens* enhanced the growth of wheat plants up to the 6% NaCl level in vitro and modulated the osmoprotectants (proline, malondialdehyde, total soluble sugar, total protein content, and indole acetic acid) to enable salt tolerance.	[71]
Root (0–150 mmol L^−1^ NaCl)	Pretreatment of *Triticum aestivum* L. seed with *Trichoderma longibrachiatum* (TG1) increased seed germination and increased the SA concentration, PAL activity, and the defense-related gene expression under saline conditions.	[72]
Root (200 mmol L^−1^ NaCl)	Highly halophilic *Bacillus* strains, isolated from the rhizosphere in the extreme environment, significantly enhanced wheat growth parameters under saline conditions.	[73]
Root (0–150 mmol L^−1^ NaCl	Saline-adapted *Bacillus pumilus* strain *JPVS11* improved the growth performance of rice (*Oryza sativa* L.) under saline conditions as well as increasing soil enzyme activities.	[74]
Root (0–200 mmol L^−1^ NaCl)	The combined effects of drought and salt stresses increased soluble sugars, leaf-free proline, carotenoid contents, and electrolyte leakage. The authors suggested that these parameters could be used as physiological indicators of drought and salinity tolerance in triticale.	[75]
Root (0–200 mmol L^−1^ NaCl)	Salt treatment significantly increased the root and shoot Na^+^ contents, and the activity of APX and GPX antioxidant enzymes, as well as increasing the relative expressions of *HvHKT2, HvHKT3, HvSOS1, HvSOS3, HvNHX1, HvNHX3, APX*, and *GPX* genes. The genotypes responded better to salinity and could be selected for the aim of releasing commercial varieties.	[76]
Leaf (250 mmol L^−1^ NaCl)	Amphidiploids of *Aegilops cylindrica*, the salt-tolerant species in the Triticeae tribe, showed significant expression patterns of the *HKT1;5, NHX1*, and *SOS1* genes associated with the exclusion of Na^+^ from the roots.	[77]
Root (0–200 mmol L^−1^ NaCl)	Trehalose application significantly increased the enzymatic activity of the trehalose metabolic pathway and increased plant biomass in tomatoes.	[78]
Root (0.200 mmol L^−1^ NaCl)	The salt-tolerant Boulifa (B) variety of barley expressed higher activities of antioxidant enzymes (SOD, APX, glutathione peroxidase, GPX, and glutathione reductase, GR) and the corresponding genes than the salt-sensitive Manzel Habib (MH) genotypes under salinity stress.	[79]

* Salinity effects through the root and leaf were primarily assessed in cereals.

**Table 2:** Growth status of plants and pathogens under different saline conditions.

Soil Salinity Class	Electrical Conductivity (μS/cm) of the Soil and the Corresponding NaCl Concentration (mmol L^−1^)		Effect on Plants	Effect on Pathogenic Microorganisms
	EC	NaCl (mmol L^−1^) *		
No detectable salt	0–2.41	0–25	The effect of salinity is negligible [168].	The effect on pathogenic microorganisms is negligible [7].
Lightly salted	2.41–4.83	25–50	Salt-sensitive plants may exhibit growth retardation [168].	Pathogenic microorganisms develop without any retardation [7].
Medium salinity	4.80–9.96	50–100	The development of many plants slows down and causes symptoms [72].	Pathogens can easily develop. Growth can be stimulated. The pathogenicity may increase [6].
Medium–high salinity	9.96–15.78	100–150	Vegetative development of crop plants is restricted. The defense system is significantly impaired [169].	Pathogens can develop. Sporulation and mycelial development are delayed, but ample sporulation is achieved [170].
High salinity (stage I)	15.78–19.61	150–200	Only tolerant plants can grow. Halophytes grow well. Other plants may produce very few flowers [168,171]	Pathogens can easily develop. Sporulation is reduced, and mycelial growth may show hyaline development [7].
High salinity (stage II)	19.61–24.56	200–250	Few tolerant plants can develop. Only halotolerant plants can grow well. Glycophytes show severe growth retardation [168].	Pathogens are stressed, and sporulation and mycelial growth decrease but continue. Stress metabolites of the pathogen tend to increase [5].
Very high salinity (stage I)	24.56–29.45	250–300	The development of salt-tolerant plants is retarded. Only obligate halotolerant plants can grow [2,168].	Sporulation and mycelial growth of pathogens are inhibited, but they can sporulate sufficiently for pathogenicity [46,171]
Very high salinity (stage II)	29.45–39.32	300–400	Growth retardation is observed in obligate halotolerant plants. Salt-tolerant glycophytes do not develop and cannot produce products [168].	The development of pathogens is severely restricted, and the infection capacity continues. Halotolerant microorganisms can easily develop [170].
Extreme salinity (stage I)	39.32–49.15	400–500	Except for obligate halophytes, plants cannot grow. Salt-tolerant glycophytes cannot grow or germinate [168].	Salt-tolerant pathogens and halotolerant microorganisms may develop, whereas other pathogens may sporulate a little. This may still be sufficient for infection [137,170].
Extreme salinity (stage II)	49.15–56.10	500–600	Obligate halophytes can develop. Almost very little vegetation survives [172,173,174].	Salt-tolerant pathogens can sporulate even if they experience significant growth retardation. Saprophytes may still develop [164,175].
Seawater–salt lake	>50	600–800	Only obligate halophytes may develop with great retardation [172,173,174].	Salt-tolerant pathogens can develop and produce high levels of stress metabolites and stress hormones. Saprophytic and salt-tolerant microorganisms experience severe growth retardation [166].
Salt lake	>50	>800	The plant cannot grow [176].	Most salt-tolerant pathogens cannot survive. Very few saprophytes show mycelial growth. Endophytes may develop. Tolerant pathogens have an infectious capacity. Mutant fungal strains may develop [176,177,178].

* Original works regarding fungal or plant growth under salinity are presented in either EC or mmol L^−1^ concentration units. We calibrated the EC and mmol L^−1^ concentration units to convert each other. This chart was prepared according to references cited in this table and the text with the help of information obtained from our previous studies. Growth conditions were considered at 25 °C. Plants under the combined stress can benefit from beneficial microorganisms thriving in a high-saline environment. Soil salinity can be reduced by both halophytes and halotolerant microorganisms. Stimulation of the plant via signaling molecules may help plants produce crops under combined stress without undergoing genetic changes. Many phylogenetically unrelated fungal species (*Cladosporium, Aspergillus, Penicillium, Emericella*, and *Eurotium* spp.) have been reported to grow at concentrations of more than 30% NaCl. A common feature of these fungi is that they can produce high levels of metabolites [176].

**Table 3:** The combined effects of salinity and pathogen stress on wheat plants and their possible improvement strategies *.

Salinity (Leaf/Root) and Pathogen Stress on Wheat	Effect on Crop Plants and Improvement Strategies **	References
Root (0–150 mmol L^−1^ NaCl) and *Fusarium pseudograminearum*	*Trichoderma longibrachiatum* (TG1) induced defenses in wheat seedlings under salinity and *Fusarium pseudograminearum* (*Fg*), a fungal agent for wheat crown rot, infection.	[72]
Root (200 mmol L^−1^ NaCl) and *Pyricularia oryzae*	Biocontrol agents (BCAs), such as *Pseudomonas stutzeri* AN10 and *Trichoderma harzianum* Th56, helped wheat plants infected with the less aggressive isolate *Pyricularia oryzae* under 200 mmol L^−1^ NaCl stress. BCAs did not enhance plant tolerance to high salt stress but improved the performance of salt-stressed wheat plants. ** The status of highly aggressive isolates remains controversial.	[46]
*Aspergillus niger*	Different priming treatments—viz., hydropriming, osmotic priming, halopriming, and hormonal priming techniques in wheat induced resistance against *Aspergillus niger* via improving the protein, proline, and sugar contents. Thaumatin-like protein (TLP), chitinase, and β-1,3-glucanase genes were highly expressed in all the primed plants.	[196]
Salinity and pathogen stress	*Leymus mollis* (Triticeae; Poaceae), a useful genetic resource for wheat (*Triticum aestivum* L.) breeding, synthesized high levels of allene oxide cyclase involved in the biosynthesis of jasmonic acid under salt stress. ** Gene transfer could be used from this plant to generate both disease-resistant and salt-tolerant wheat plants.	[197]
Salinity and pathogen stress	*Cakile maritima*, a halophytic plant, tolerated the pathogenic attack of *Alternaria alternate* under 200 mmol L^−1^ NaCl conditions. ** However, this is not always the case if plants cannot tolerate the high level of salinity.	[198]
Abiotic and pathogen stress	miRNA-mediated modulation of *AtP5CS1* gene expression under combined stress filled crucial gaps.	[199]
Abiotic and pathogen stress	Silicon and hydrogen sulfide improved the physiological resistance of wheat plants to drought stress and the pathogen *Puccinia tritici* infection. Drought stress increased the susceptibility of wheat plants to leaf rust pathogen infection.	[200]
*Z. tritici* stress on wheats	*Trichoderma harzianum* reduced pycnidia coverage of the pathogen in the susceptible cultivar. ** Salt-tolerant *T. harzianum* or other species could be used under both saline and pathogen conditions.	[201]
Salinity on wheat	Effective microorganisms (EMs) could be used to remediate the saline soils growing wheat. ** Promising EM species controlling *Z. tritici* could be very useful to improve the conditions of wheat under the combined stress.	[53]
*Z. tritici* stress on wheat	*Thinopyrum elongatum* is suggested for use since it contains important genetic resources. It carries genes resistant to *Fusarium*, *Zymoseptoria*, and rust diseases as well as tolerance genes to soil salinity, low temperatures, water logging, etc., and some quality traits for bakery.	[202]

* Due to difficulties in finding specific articles directly related to the wheat–*Z. tritici*–salinity stress model, we selected some articles on the topic of wheat affected by other pathogens under salinity stress conditions, as well as research on other plants infected by leaf pathogens under saline conditions. With the help of this information, we aim to draw conclusions and evaluate the combined stress mechanism and potential improvement strategies for crop plants. ** Our comments.
